# Disparities in disease presentation and survival after pathological fracture surgery at a middle- and a high-income centre

**DOI:** 10.1186/s12893-026-03690-w

**Published:** 2026-03-30

**Authors:** Martina Byttner, Panagiotis Tsagkozis, Rikard Wedin, Henrik C. F. Bauer, Thomas Hilton

**Affiliations:** 1 Department of Trauma and Reparative Medicine, Karolinska University Hospital, Stockholm, Sweden; 2 Department of Molecular Medicine and Surgery, Karolinska Institute, Stockholm, Sweden; 3 Department of Surgery, Division of Orthopaedic Surgery, Faculty of Medicine and Health Science, Groote Schuur Hospital, University of Cape Town, Cape Town, South Africa

**Keywords:** Metastatic bone disease, Pathological fracture, Fractures, bone/pathology, Bone neoplasms/secondary, Survival outcomes, Health care disparities, Developing countries, Africa South of the Sahara

## Abstract

**Background:**

Cancer prevalence is rapidly increasing in low- and middle-income countries, where late diagnosis and limited healthcare resources contribute to poor outcomes. The aim of this study was to compare presentation patterns and survival outcomes between Groote Schuur Hospital (South Africa) and Karolinska University Hospital (Sweden) to highlight disparities between high- and middle-income healthcare settings.

**Method:**

This retrospective cohort study used data from the International Bone Metastasis Registry. Patients who underwent surgery for pathological fractures between 2018 and 2023 were included. The primary objective was to compare the proportion of patients presenting with malignancy not diagnosed prior to surgery between the two centres. Secondary outcomes included newly diagnosed malignancy within two months from surgery, indication for surgery, ECOG performance status, and survival.

**Results:**

A total of 362 patients were included; 107 from Groote Schuur Hospital (GS) and 255 from Karolinska University Hospital (KS). GS had a higher proportion of patients with malignancy not diagnosed prior to surgery (13% vs 4%; *p =* 0.004) and newly diagnosed malignancy within two months (50% vs 25%; *p <* 0.001). GS patients were younger, more often female, and more frequently had breast cancer. They had fewer visceral metastases and better ECOG performance status. Prophylactic fixation was more common at GS, while surgical techniques were similar. No statistically significant difference in postoperative survival from surgery was detected between centres (*p =* 0.065); however, median postoperative survival was longer at GS (13 months) compared with KS (6 months), and the proportion of long-term survivors (> 2 years) was higher at GS (17% vs 3%; *p <* 0.001). Median overall survival from cancer diagnosis was longer at KS (247 months) compared with GS (43 months; *p <* 0.001).

**Conclusions:**

Patients at GS were more likely to present with previously undiagnosed or newly diagnosed malignancy, reflecting delayed detection and referral. They had better performance status, fewer visceral metastases, and more frequent prophylactic surgery. Despite similar surgical methods, postoperative survival did not differ significantly. These findings underscore the need for earlier cancer detection and improved access to systemic therapy in resource-limited settings.

**Supplementary Information:**

The online version contains supplementary material available at 10.1186/s12893-026-03690-w.

## Introduction

The prevalence of cancer is increasing globally and even more so in low- and middle-income countries, with outcomes significantly worse than those observed in high-income nations [1]. In 2018, 18 million new cancer cases were reported globally, projected to reach 40 million annually by 2040 [2]. Metastasis frequently involves the skeleton, with about 5% of cancer patients being diagnosed with metastases to the bone each year [3]. Depending on primary cancer type, patient age, patient performance status, presence of visceral metastases, and extent of bone involvement, about 50–70% of patients will die within one year of their pathological fracture [4, 5]. Palliative surgical and oncological treatment is often recommended to alleviate pain and to improve function, thereby enabling patients to remain at home and out of hospital.

It is important to identify patients with very limited expected survival and patients who can survive several years, as the choice of reconstruction can be quite different between these groups [6, 7]. This has important implications for both patient morbidity and healthcare resource allocation.

In South Africa, cancer diagnoses often occur at an advanced stage, resulting in poorer survival outcomes compared to high-income countries [8–10]. However, data on patients with pathological fractures and their outcomes in South Africa remain limited, highlighting a critical gap in knowledge and care. The International Bone Metastasis Registry (IBMR) was initiated in 2015 and contains longitudinally collected data of bone metastasis patients from 10 countries. Since 2018, data from Groote Schuur hospital in Cape Town, South Africa are included as the first African country that contributes to IBMR.

While several studies from high-income countries have described the presentation, management, and outcomes of patients with metastatic bone disease, comparative studies between low- and middle-income and high-income settings are lacking [5–7]. To our knowledge, no prior study has systematically compared patients undergoing surgery for pathological fractures between a low- and middle-income country (LMIC) and a high-income country (HIC) context..

The aim of this study was to compare patterns of cancer presentation, disease characteristics, and surgical outcomes in patients undergoing surgery for pathological fractures at Karolinska University Hospital (KS) and Groote Schuur Hospital (GS). The primary objective was to compare the proportion of patients presenting with malignancy not diagnosed prior to surgery between the two centres. Secondary objectives included differences in extent of disease, demographic characteristics, surgical management, and survival outcomes.

### Setting

GS is a major public academic hospital in Cape Town, serving as both a primary and tertiary referral centre for the Western Cape Province as well as for other regions of South Africa. Healthcare is publicly funded and provided at low or no cost to most patients, although the system faces significant challenges related to resource constraints, healthcare disparities, and social determinants of health.

KS in Stockholm is one of Sweden’s largest academic hospitals, providing advanced healthcare services and serving as a tertiary referral centre. The hospital benefits from well-established diagnostic infrastructure and a comprehensive, publicly funded healthcare system.

Healthcare delivery and diagnostic pathways differ between the two settings. Access to population-based cancer screening, timely advanced diagnostic imaging, radiotherapy, and modern systemic therapies is more readily available in the Swedish healthcare system, whereas patients treated in the South African public healthcare system may experience longer diagnostic and referral pathways due to resource constraints. These system-level differences should be considered when interpreting differences in disease presentation and outcomes between the two centres.

### Patients and methods

This retrospective cohort study used data from the International Bone Metastasis Registry (IBMR). Data from all patients were prospectively and consecutively entered into the registry by designated clinicians at each centre. Radiological, pathological, and biochemical data were obtained from the electronic patient records. Performance status was classified according to ECOG criteria based on documented functional status in the medical records. Data completeness and internal consistency were reviewed at the time of entry.

Patients were eligible if they underwent surgery for impending or completed pathological fractures of the appendicular skeleton or pelvis caused by metastatic bone disease. All patients treated surgically for pathological fractures at the tertiary referral centres GS and KS between 01/01/2018 to 31/12/2023 were included. Patients with spinal fractures, or non-surgically treated fractures (such as ribs or sternum) were excluded. A minimum follow-up period of two months was required for surviving patients.

### Outcomes and variables

The primary outcome was the proportion of patients presenting with a previously undiagnosed malignancy at the time of surgery, defined as those who had not previously been diagnosed with cancer and had no biopsy-proven or otherwise identified primary tumour at the time of surgery.

One secondary outcome was the proportion of patients with a newly diagnosed malignancy within two months of surgery. A newly diagnosed malignancy was defined as a first cancer diagnosis made during preoperative assessment or within two months after surgery, based on biopsies or radiological investigations performed shortly before or after surgery for the pathological fracture.

These outcomes were chosen to enable comparison of diagnostic and referral pathways between the two healthcare settings, as unknown primary tumour or newly diagnosed malignancy may reflect delayed cancer diagnosis or late presentation.

Other secondary outcomes included the extent of oncological disease at the time of surgery, classified as single bone metastasis, multiple bone metastases, or the presence of visceral metastases. For patients with a known cancer diagnosis, staging was based on the most recent information available in the medical record, without additional preoperative restaging. For patients with newly diagnosed malignancy, staging was based on available diagnostic imaging and clinical investigations performed during the diagnostic work-up.

Additional factors included performance status assessed using the Eastern Cooperative Oncology Group (ECOG) classification, demographic data (such as sex, age at surgery, and location of pathological fracture), and surgical details (indication for surgery, type of reconstruction, and implants used).

Statistical analysis was carried out in SPSS (version 25, SPSS Inc, Chicago, IL). Categorical variables were compare using the Pearson’s chi-square (χ^2^) test and continuous variables using the Mann–Whitney U test. Survival analysis was done according to the Kaplan–Meier method and the log-rank test was used for comparisons between groups. All tests were double-sided, and a *p* value of ≤ 0.05 was considered significant.

## Results

A total of 362 patients were included, 255 from KS and 107 from GS. Demographic details and an overall description of the cohort are presented in Table 1.

**Table 1:** Baseline characteristics of patients treated at Groote Schuur Hospital (GS) and Karolinska Hospital (KS)

	**GS (*n* = 107)**	**KS (*n* = 255)**	***p*-value**
Gender, no. of patients (%)			0.004
Female	70 (65)	125 (49)	
Male	37 (35)	130 (51)	
Age at surgery, median (years)	60	72	< 0.001
ECOG performance status, no. of patients (%)*			< 0.001
0–1	54 (51)	40 (16)	
2–4	32 (30)	191 (75)	
Unknown	21 (20)	24 (9)	
Metastatic load, no. of patients (%)			< 0.001
Single bone metastasis	19 (18)	18 (7)	
Multiple bone metastases	59 (55)	86 (34)	
Visceral metastases	26 (24)	148 (58)	
Unknown	3 (3)	3 (1)	

Values are given as number (%) unless otherwise stated

*Abbreviations*: *GS* Groote Schuur Hospital, *KS* Karolinska Hospital, *ECOG* Eastern Cooperative Oncology Group performance status

^*^ECOG *p*-value calculated comparing ECOG 0–1 versus ECOG 2–4; patients with missing ECOG data were excluded from the statistical test

### Demographic characteristics

Patients at GS were younger, with a median age of 60 years compared to 72 years at KS (*p <* 0.001). Women were more prevalent in the GS cohort (65% vs. 49%, *p =* 0.004). This difference corresponded to a variation in the distribution of primary diagnoses (*p =* 0.001), with breast cancer being the most common cancer type and twice as prevalent at GS (36% vs. 19%). In contrast, prostate cancer was twice as common in Sweden (10% vs. 4%). The distribution of primary cancer sites is described in Table 2.

**Table 2:** Primary cancer site and localization of pathological fracture

	**GS (*n* = 107)**	**KS (*n* = 255)**	**Total (*n* = 362)**	***p*-value**
Primary cancer site, no. of patients (%)				**0.001**
Breast	39 (36%)	48 (19%)	87 (24%)	
Myeloma	17 (16%)	41 (16%)	58 (16%)	
Lung	18 (17%)	36 (14%)	54 (15%)	
Kidney	9 (8%)	31 (12%)	40 (11%)	
Prostate	4 (4%)	26 (10%)	30 (8%)	
Lymphoma	4 (4%)	12 (5%)	16 (4%)	
Melanoma	1 (1%)	9 (4%)	10 (3%)	
Bladder	2 (2%)	9 (4%)	11 (3%)	
Other	10 (9%)	36 (14%)	46 (13%)	
Unknown	3 (3%)	7 (3%)	10 (3%)	
Localization of pathological fracture, no. of patients (%)				**0.2**
Femur	79 (74%)	161 (63%)	240 (66%)	
Humerus	11 (10%)	60 (24%)	71 (20%)	
Pelvis	12 (11%)	23 (9%)	35 (10%)	
Other	5 (5%)	11 (4%)	16 (4%)	

*Abbreviations*: *GS* Groote Schuur Hospital, *KS* Karolinska Hospital, *No.* Number of

At surgery, GS patients had better performance status and less extensive disease, reflected by fewer visceral metastases (25% vs 59%; *p <* 0.001) and a higher proportion with ECOG 0–1 (51% vs 16%; *p <* 0.001).

### Patients without a prior cancer diagnosis and overall survival

The proportion of patients with malignancy not yet diagnosed at the time of surgery was three times more common at GS than at KS (13% vs. 4%, *p =* 0.004). Similarly, the proportion of patients newly diagnosed with a malignancy within two months of surgery was twice as common at GS compared to KS (50% vs. 25%, *p <* 0.001).

In this unadjusted analysis, no statistically significant difference in post-operative survival was detected between the two centres (*p =* 0.065), although GS patients showed a trend towards longer postoperative survival, particularly within the first two years after surgery.

**Fig. 1:**
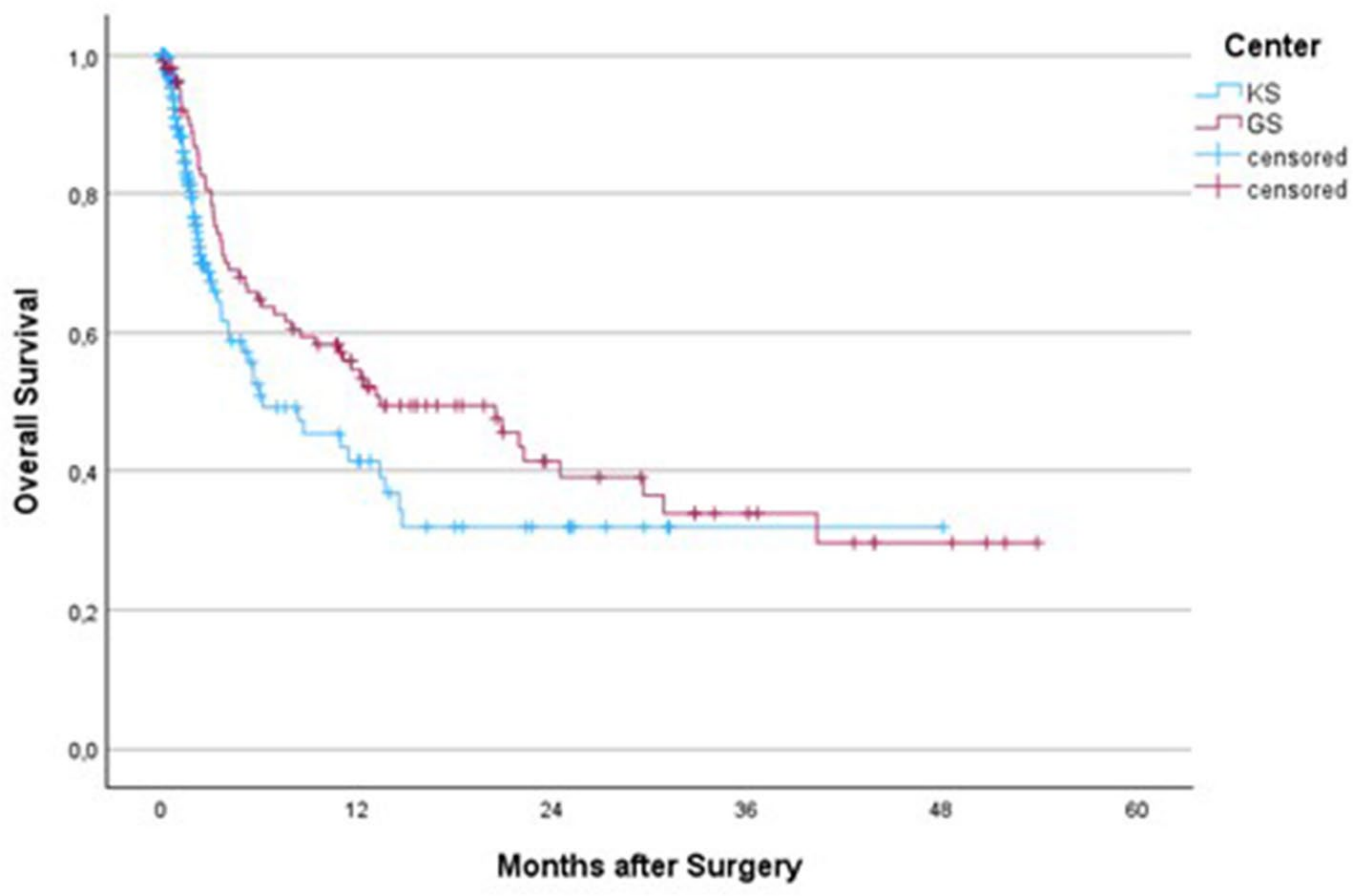
Overall survival from pathological fracture. Log-rank *p =* 0.065

Median postoperative survival was longer for GS patients (13 months) compared with KS patients (6 months; *p =* 0.065). Similarly, the proportion of long-term survivors (> 2 years) was higher at GS (17% vs 3%; *p <* 0.001). This pattern is illustrated in Fig. 1.

**Fig. 2:**
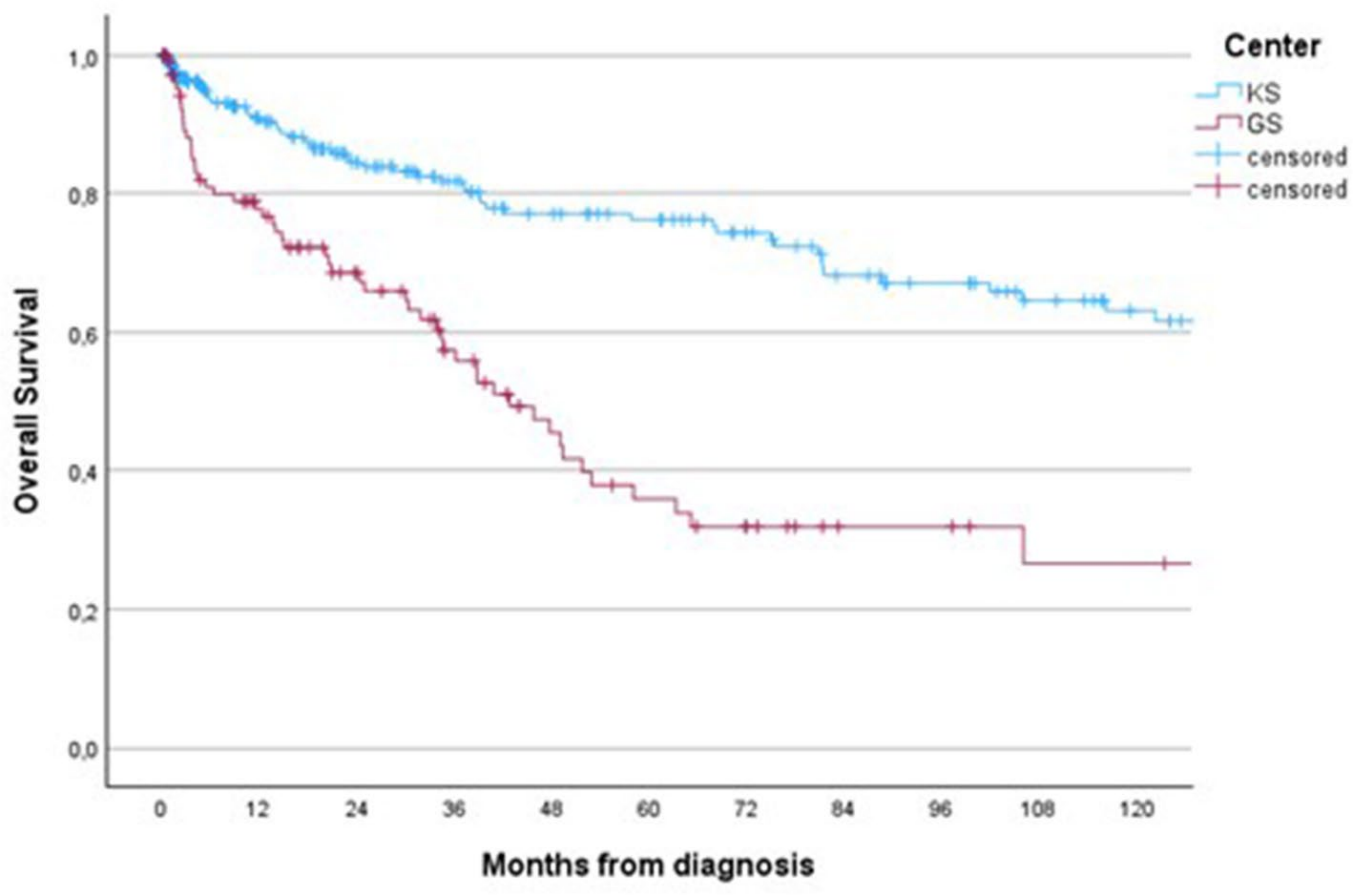
Overall survival from the time of diagnosis. Log-rank *p <* 0.001

When stratified by indication for surgery, no statistically significant difference in survival was detected between centres among patients operated prophylactically for impending fractures (log-rank *p =* 0.53), whereas those operated for completed pathological fractures had significantly longer survival at GS (log-rank *p =* 0.04; Supplementary Figure S1). Overall survival from the time of diagnosis differed between the two cohorts (log-rank *p <* 0.001; Fig. 2). Median survival from diagnosis was 43 months in GS compared with 247 months in KS.

### Modes of surgical treatment

**Fig. 3:**
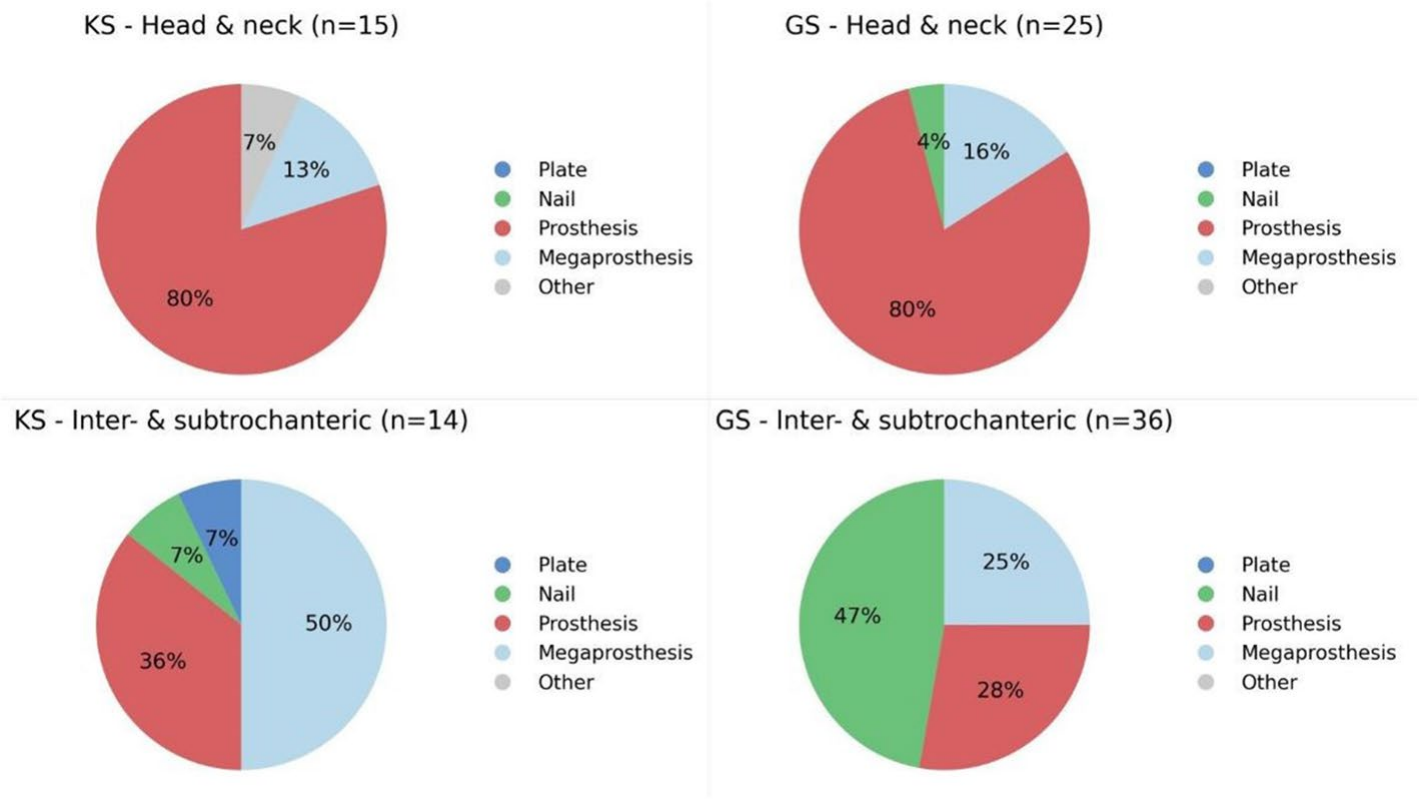
Treatment methods of the proximal femur

The indication for surgical treatment varied significantly. In the GS cohort, half of the patients were operated for what was considered impending fractures, whereas in the KS cohort, only 25% of patients were operated to prevent a pathological fracture (*p <* 0.001). There was no significant difference in the site of pathological fractures between the two centres (*p =* 0.2, Table 2). The surgical methods and types of implants used were similar across the two cohorts (Fig. 3).

The proximal femur was the most common fracture localization. Prosthetic reconstruction was the preferred treatment for fractures in the inter- and subtrochanteric regions at both centres, more commonly used at KS (86%) than at GS (53%). Conversely, intramedullary (IM) nailing was performed in 47% at GS and only in 7% at KS (*p =* 0.009). This difference in implant choice was influenced by the higher proportion of patients with impending fractures in the GS cohort.

**Fig. 4:**
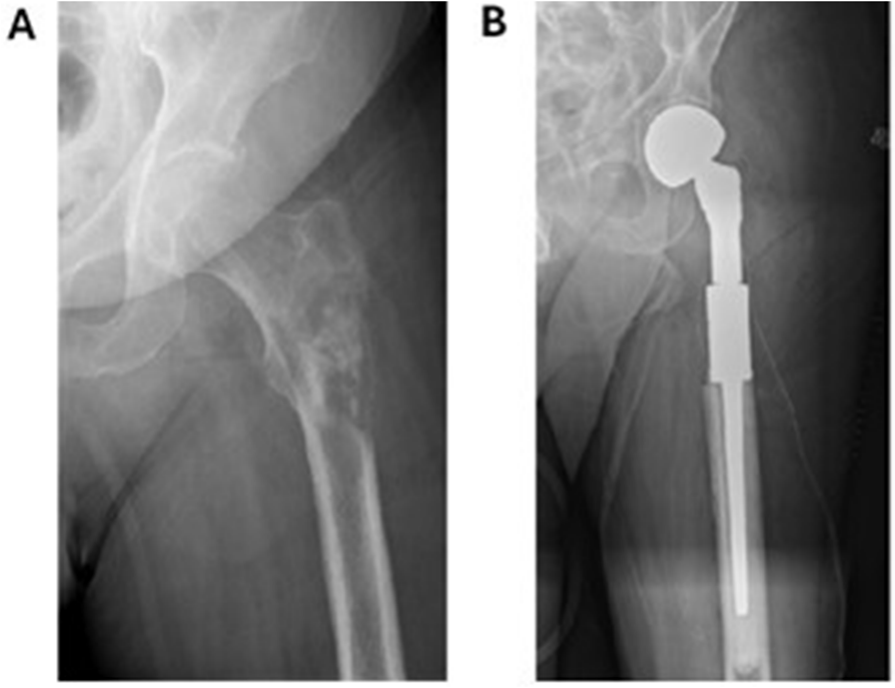
Preoperative and postoperative imaging of a 65-year-old patient with generalized prostate cancer treated at KS. **A** 65-year-old man with a 7-year history of prostate cancer and known skeletal and visceral mestastases. He represents with increasing pain in the femur. Radiographs demonstrate a pathological fracture through a known femoral metastasis. **B** The patient underwent resection of the proximal femur and reconstruction with a cemented proximal femoral megaprosthesis

**Fig. 5:**
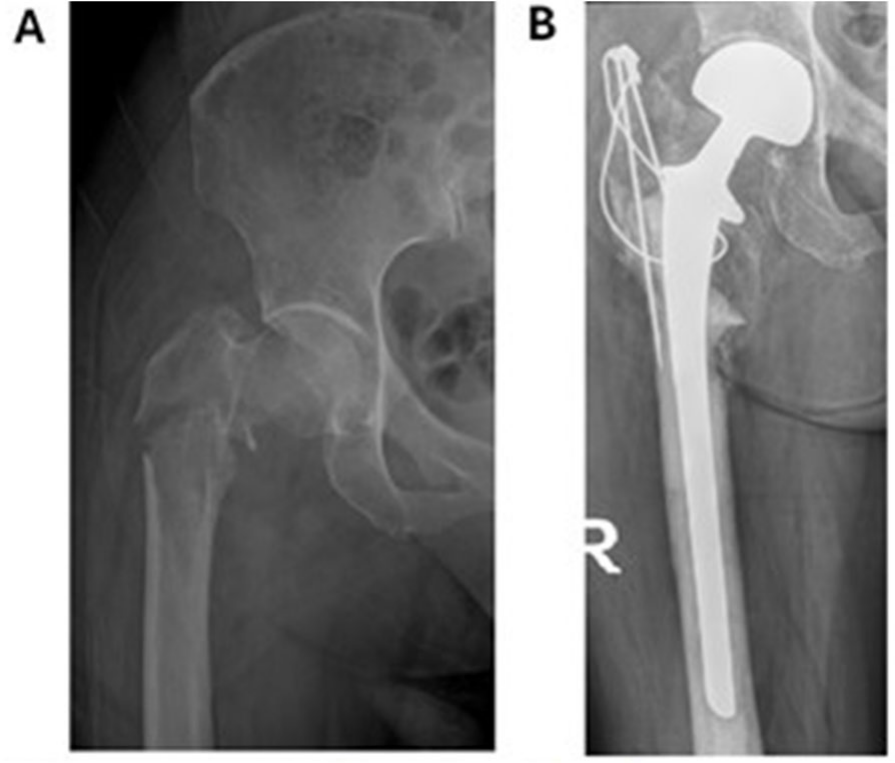
Preoperative and postoperative imaging of a patient with previously unknown breast cancer treated at GS. **A** 58 years old healthy woman admitted after fall. Radiographs showed pathological fracture and clinical examination an ulcerated, fungating 3 cm tumour of the left breast. Core biopsy of breast tumour showed breast cancer. **B** The patient underwent resection of proximal femur and recunstruction with a cemeted, long-stem hemiarthropalsty. Histology of surgical specimen confirmed metastasis of breast cancer. CT staging study performed 3 months after diagnosis revealed no other signs of metastases

For fractures of the femoral head and neck, prosthesis was the primary treatment for most patients (93% at KS and 96% at GS). Figures 4 and 5 illustrates typical surgical reconstructions for proximal femoral fractures.

## Discussion

This study provides insights into differences in cancer presentation, management, and survival among patients undergoing surgery for metastatic bone disease in two tertiary referral centres operating in contrasting healthcare settings. As hypothesised, patients at Groote Schuur Hospital (GS) more often presented with pathological fractures without a prior cancer diagnosis compared with patients at Karolinska University Hospital (KS).

Survival comparisons between the two centres were based on unadjusted analyses, and the cohorts differed in baseline characteristics, including performance status, disease burden, and indication for surgery. Despite presenting with better performance status and fewer visceral metastases, no statistically significant difference in postoperative survival was detected between GS and KS. This finding suggests that short-term surgical outcomes may be influenced less by preoperative condition and more by systemic factors such as access to oncologic treatment, continuity of care, and follow-up.

The high proportion of patients at GS presenting with previously undiagnosed or newly diagnosed malignancy likely reflects delays in cancer detection and referral for definitive treatment. Late-stage cancer diagnosis remains a persistent challenge, largely due to limited access to healthcare, diagnostic tools, and early detection programs [11]. Social factors further exacerbate these delays. Many patients reside in remote areas without reliable transportation or financial resources for travel [12, 13]. Family responsibilities and other pressing issues are often prioritized over seeking medical attention for a suspected cancer, especially among female patients who traditionally shoulder all of the domestic responsibilities [14]. Additionally, fear, stigma, traditional beliefs, and limited awareness about the importance of early detection contribute to postponing treatment [11, 14, 15]. Consequently, 54% of breast cancer patients in South Africa presented with a more advanced disease, significantly reducing treatment options and worsening outcomes [8–10].

Demographic differences between cohorts, including younger age and a higher proportion of women in the GS group, particularly those with breast cancer, likely reflect variations in cancer epidemiology and access to care. Breast cancer is the most common malignancy among women in both South Africa and Sweden, but lifetime risk and outcomes differ considerably. In South Africa, cumulative risk varies significantly between population groups (women of European ancestry 9.6%; women of African ancestry 2.8%), compared with 10.4% in Sweden [16, 17]. Five-year survival rates are markedly higher in Sweden than in South Africa (93% versus 40%) [16, 18].

The higher proportion of breast cancer with de novo bone metastases at GS may reflect a combination of biological and systemic factors. More aggressive breast cancer subtypes, such as triple-negative breast cancer, are more prevalent in parts of sub-Saharan Africa and have been associated with earlier metastatic spread [8, 19]. However, these tumours typically metastasise preferentially to visceral organs rather than to bone [20, 21]. However, the lower prevalence of visceral metastases and better performance status in GS patients suggests that tumour biology alone is unlikely to explain the observed survival patterns.

In South Africa, mammography participation remains low, further hindering early diagnosis and timely intervention [22, 23]. Notably, GS patients had better functional status and fewer visceral metastases at the time of surgery compared with KS patients. This likely reflects that KS patients typically present with pathological fractures later in their disease course, after multiple lines of systemic therapy, whereas GS patients more often present de novo with Stage IV disease without prior oncologic treatment. As a result, GS patients underwent surgical intervention earlier in their disease trajectory, before the cumulative burden of prolonged cancer treatment had affected their general condition.

Although both centres serve as tertiary referral hospitals, more than half of the GS patients underwent surgery for impending fractures, compared to only a quarter in KS. This disparity likely reflects differences in healthcare priorities and system capacity. In South Africa, surgical fixation of impending fractures may be favoured due to constraints such as more limited access to radiotherapy, compressed operating theatre availability, and concerns about reliable follow-up [24, 25]. This often results in a “one-chance” treatment approach. The longer postoperative survival observed among GS patients, particularly among those treated for completed fractures, is more likely to reflect differences in timing and clinical context at presentation than true prognostic differences.

Although operating theatre access is constrained, access to radiotherapy is even more limited at GS. This is consistent with national-level data from the International Atomic Energy Agency’s Directory of Radiotherapy Centres (DIRAC), which indicate substantially lower radiotherapy capacity per capita in South Africa compared with Sweden [26]. Pathological fractures with less than 50% cortical involvement are also rarely seen in the GS cohort, further supporting the decision to stabilise surgically when possible. In contrast, the “wait-and-see” approach observed in Sweden is supported by greater capacity for imaging follow-up, consistent monitoring, and access to non-surgical interventions. Radiotherapy remains an important component of metastatic bone disease management, particularly for symptom relief and local tumour control. Its broader availability at KS allows it to be incorporated more routinely into treatment pathways.

Despite these differences, surgical methods and implant selection were comparable between centres, indicating similar technical standards of care. Intramedullary nailing was more common at GS, reflecting the higher proportion of prophylactic stabilisations. Both centres clearly favour prosthetic replacement over plate fixation. However, this may not be the case in non-university hospitals with more limited reconstructive options.

Overall post-operative survival did not differ significantly between the two centres, but outcomes differed when considering surgical indication. GS patients operated for completed pathological fractures tended to have better short-term survival, whereas outcomes for prophylactic fixation were similar between centres. The higher proportion of long-term survivors in GS was likely related to less extensive systemic involvement and general condition at the time of surgery. However, when survival was measured from the time of diagnosis, KS patients had better outcomes, likely due to earlier detection and comprehensive oncological care. Together, these findings reinforce the importance of early diagnosis, equitable access to oncologic care, and timely multidisciplinary intervention in improving overall survival outcomes.

### Limitations

This study has limitations. Some potentially relevant prognostic variables and details on systemic and adjuvant treatments were not consistently captured, and certain variables contained missing data. These factors may have influenced treatment decisions and outcomes. In addition, differences in diagnostic and referral pathways prior to hospital presentation could not be fully accounted for and may have contributed to observed differences between cohorts. Finally, as this was a retrospective study conducted at two tertiary academic centres, the findings may not be fully generalisable to other healthcare settings.

### Implications for resource allocation and treatment planning

The findings of this study highlight the need for improved referral pathways and earlier cancer detection at GS. Earlier cancer detection may allow for more effective oncologic management and reduce the number of patients presenting with pathological fractures without prior cancer diagnosis. Strengthening public health initiatives, improving access to diagnostic tools, and reinforcing primary care services are essential to address delays in cancer diagnosis.

At the same time, the higher proportion of impending fractures treated at GS likely reflects a clinical need for early stabilisation, considering challenges in follow-up and resource availability. Despite these constraints, the comparable surgical practices between the two centres demonstrate that high-quality surgical care for metastatic bone disease can be delivered in resource-limited settings. Efficient use of available resources and better coordination of care will be essential to improve treatment quality and outcomes.

## Conclusion

This study underscores substantial disparities in the timing of cancer diagnosis, disease presentation, and referral pathways between two tertiary centres in South Africa and Sweden. Patients at Groote Schuur Hospital (GS) more frequently presented with pathological fractures without a prior cancer diagnosis or were newly diagnosed at the time of surgery, reflecting delays in diagnosis and referral.

Despite having better performance status and fewer visceral metastases, GS patients had similar postoperative survival to those at Karolinska University Hospital (KS), suggesting that factors beyond surgical care, including access to systemic therapy and coordinated long-term care, influence long-term outcomes. Differences in overall survival from diagnosis highlight the importance of earlier detection and access to comprehensive cancer care. Addressing these disparities through better integration of cancer care and improved access to systemic therapy is essential for improving outcomes for patients in South Africa and other low- and middle-income countries.

## Supplementary Information

Supplementary Material 1.

## Data Availability

Individual de-identified participant data will be made available upon reasonable request from the corresponding author.
