# Supplementary figures and images for: Disparities in disease presentation and survival after pathological fracture surgery at a middle- and a high-income centre
Source: BMC Surg. 2026 Mar 30;26:255. doi: 10.1186/s12893-026-03690-w (PMC13063899; doi:10.1186/s12893-026-03690-w)

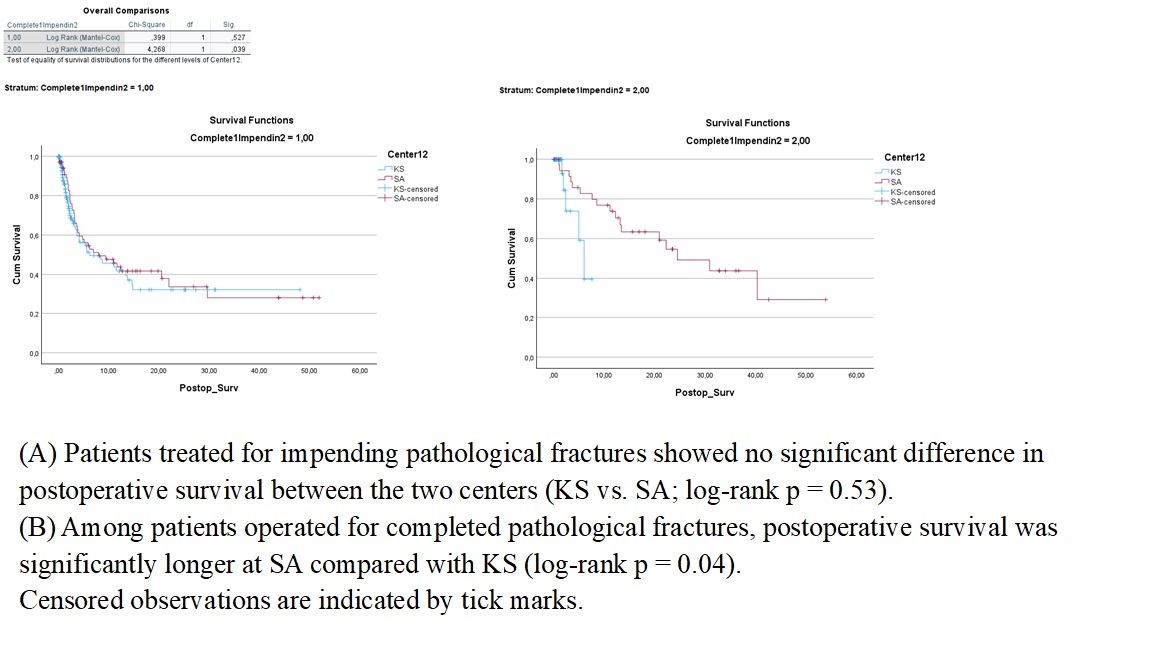

Supplement: Supplementary file 1 — Supplementary Material 1. [file 12893_2026_3690_MOESM1_ESM.jpg]
